# Microbiome of Deep Dentinal Caries Lesions in Teeth with Symptomatic Irreversible Pulpitis

**DOI:** 10.1371/journal.pone.0154653

**Published:** 2016-05-02

**Authors:** Isabela N. Rôças, Flávio R. F. Alves, Caio T. C. C. Rachid, Kenio C. Lima, Isauremi V. Assunção, Patrícia N. Gomes, José F. Siqueira

**Affiliations:** 1 Department of Endodontics and Molecular Microbiology Laboratory, Estácio de Sá University, Rio de Janeiro, RJ, Brazil; 2 Institute of Microbiology Prof. Paulo de Góes, Federal University of Rio de Janeiro, RJ, Brazil; 3 Department of Preventive Dentistry, Federal University of Rio Grande do Norte, Natal, RN, Brazil; National Cancer Institute, UNITED STATES

## Abstract

This study used a next-generation sequencing approach to identify the bacterial taxa occurring in the advanced front of caries biofilms associated with pulp exposure and irreversible pulpitis. Samples were taken from the deepest layer of dentinal caries lesions associated with pulp exposure in 10 teeth diagnosed with symptomatic irreversible pulpitis. DNA was extracted and the microbiome was characterized on the basis of the V4 hypervariable region of the 16S rRNA gene by using paired-end sequencing on Illumina MiSeq device. Bacterial taxa were mapped to 14 phyla and 101 genera composed by 706 different OTUs. Three phyla accounted for approximately 98% of the sequences: Firmicutes, Actinobacteria and Proteobacteria. These phyla were also the ones with most representatives at the species level. Firmicutes was the most abundant phylum in 9/10 samples. As for genera, *Lactobacillus* accounted for 42.3% of the sequences, followed by *Olsenella* (13.7%), *Pseudoramibacter* (10.7%) and *Streptococcus* (5.5%). Half of the samples were heavily dominated by *Lactobacillus*, while in the other half lactobacilli were in very low abundance and the most dominant genera were *Pseudoramibacter*, *Olsenella*, *Streptococcus*, and *Stenotrophomonas*. High bacterial diversity occurred in deep dentinal caries lesions associated with symptomatic irreversible pulpitis. The microbiome could be classified according to the relative abundance of *Lactobacillus*. Except for *Lactobacillus* species, most of the highly prevalent and abundant bacterial taxa identified in this study have been commonly detected in infected root canals. The detected taxa can be regarded as candidate pathogens for irreversible pulpitis and possibly the pioneers in pulp invasion to initiate endodontic infection.

## Introduction

Caries is the major cause of pulp inflammation [1]. As soon as the caries biofilm destroys enamel and reaches dentin, subtle inflammatory changes are already evident near the pulp border adjacent to the affected dentinal tubules [2–3]. As the caries lesion progresses deep in dentin and towards to the pulp, the severity of pulp inflammation increases [4]. When the pulp tissue is frankly exposed to the caries biofilm, a severe inflammatory reaction develops, often resulting in localized abscesses. At this point, pulpitis is regarded as irreversible in the sense that only removal of the cause (caries) does not suffice to promote pulp healing [5–6]. The condition is not always painful; actually, irreversible pulpitis is usually asymptomatic [7]. When present, symptoms can vary in intensity and duration; in some cases, pain can be spontaneous, severe, diffuse, and difficult to control with analgesics, requiring emergency treatment [8].

The microbiota associated with dentinal caries has been studied by culture [9–11] and molecular microbiology methods [12–14]. Overall, findings from these studies have shown distinct bacterial community composition in comparison to enamel lesions. Lactobacilli have been reported to prevail in dentinal caries [10, 12, 14–15], but species/phylotypes of the genera *Prevotella*, *Selenomonas*, *Dialister*, *Fusobacterium*, *Atopobium*, *Olsenella*, *Bifidobacterium*, and *Pseudoramibacter* have also been commonly identified [9, 11–14, 16–18].

Recently, next-generation DNA sequencing (NGS) methods have been applied to the study of the microbiome of dentinal caries biofilms. A study combined culture, clone library and Sanger sequencing, and pyrosequencing (a NGS approach) to analyze 6 samples from dentinal caries [19] and reported the dominance of *Prevotella*, *Lactobacillus*, *Selenomonas*, and *Streptococcus* representatives. Two samples were dominated by lactobacilli, while the others had significantly high levels of *Prevotella* species. Pyrosequencing revealed a large bacterial diversity; over 260 species-level taxa were detected. A study also used pyrosequencing to investigate the shifting bacterial profiles associated with different caries states in children and observed reduced bacterial diversity in dentinal caries in comparison to the other sites [20]. The genera *Cryptobacterium*, *Lactobacillus*, *Megasphaera*, *Olsenella*, *Scardovia*, *Shuttleworthia*, *Cryptobacterium*, and *Streptococcus* were in significantly increased levels in dentinal caries samples. Another pyrosequencing study evaluated the microbiome of dentinal caries layers ranging in pH from 4.5 to 7.8 and reported the presence of representatives from 9 phyla, 97 genera and over 400 species [21]. The most abundant taxa included representatives of the genera *Lactobacillus*, *Prevotella*, *Atopobium*, *Olsenella* and *Actinomyces*. The microbiota associated with acidic conditions showed lower diversity and was dominated by *Lactobacillus* species.

Although several studies have evaluated the dentinal caries microbiota, there is still limited information as to the bacterial taxa associated with still more advanced caries lesions, e.g., in teeth with pulp exposure and irreversible pulpitis. It has been shown that the cultivable microbiota of cariously exposed pulps was similar in composition to that of carious biofilms, except that fewer species/taxa were identified in the pulp samples [22]. Studies have shown that species from the genera *Prevotella*, *Porphyromonas*, *Streptococcus*, *Parvimonas*, *Actinomyces*, *Dialister* and *Fusobacterium* present in deep dentinal lesions were associated with pulp symptoms [10, 18, 23–25]. In a previous study using a closed-ended molecular microbiology technique (reverse-capture checkerboard), we examined samples from advanced dentinal caries lesions associated with irreversible pulpitis and reported that the most frequent taxa were *Atopobium* genomospecies C1, *Pseudoramibacter alactolyticus*, *Streptococcus* species, *Parvimonas micra*, *Fusobacterium nucleatum* and *Veillonella* species [25].

The present study used a NGS approach to characterize the microbiome occurring at the forefront layer of dentinal caries and associated with pulp exposure in teeth with symptomatic irreversible pulpitis. By using an open-ended molecular microbiology approach, this study is expected to contribute to the knowledge of the bacterial taxa involved with irreversible pulp inflammation.

## Materials and Methods

### Case selection and sample taking

Samples were taken from deep occlusal caries in permanent molars from 10 individuals (6 female and 4 male) ranging in age from 16 to 60 years (mean, 29.9 years). Included teeth had dentinal caries lesions that resulted in pulp exposure and were diagnosed with symptomatic irreversible pulpitis on the basis of clinical and radiographic findings, according to the reports of the American Association of Endodontists Consensus Conference on diagnostic terminology [5]. Pulp vitality was confirmed by thermal sensibility tests. Radiographic analysis showed extensive caries lesions, mature root apexes, and normal width of the apical periodontal ligament space. Teeth presenting with necrotic pulps or treated root canals, as well as teeth with no evidence of pulp exposure following caries removal, were excluded from the study. Individuals exhibited no evidence of marginal periodontitis and reported no significant systemic condition. The study protocol was approved by the Ethics Committee of the Federal University of Rio Grande do Norte and informed consent was obtained from all individuals or their parents.

For sample taking, each tooth was initially cleaned with pumice and, under local anesthesia, the undermined enamel, debris and the most superficial layer of the caries lesion were removed by using high speed burs under copious water cooling. Rubber dam was applied and the operative field was cleaned and disinfected with 6% hydrogen peroxide and 2.5% sodium hypochlorite, respectively. Next, 10% sodium thiosulfate was applied to neutralize sodium hypochlorite and a sterile spoon excavator was used to remove the dentinal caries layers until the deepest layer, covering the pulp, was left. Another sterile excavator was used to collect this deepest layer of dentinal caries contacting the pulp. If after removal of the last caries layer no pulp exposure was evident, the tooth was excluded from the study. The sample material was placed in cryotubes containing Tris-EDTA buffer (10 mM Tris-HCl, 1 mM EDTA, pH 7.8) and immediately frozen at -20°C.

### 16S rRNA gene sequencing and bioinformatic analysis

DNA was extracted from caries dentin samples by using the QIAamp DNA Mini Kit (Qiagen, Valencia, CA, USA), following the protocol for tissues as recommended by the manufacturer.

The 16S rRNA gene V4 variable region primers 515/806 [26] with barcode on the forward primer were used in a 30-cycle polymerase chain reaction (PCR) assay using the HotStarTaq Plus Master Mix Kit (Qiagen) under the following conditions: 94°C for 3 minutes, followed by 28 cycles of 94°C for 30 seconds, 53°C for 40 seconds and 72°C for 1 minute, and then a final elongation step at 72°C for 5 minutes. After amplification, PCR products were checked in 2% agarose gel to determine the success of amplification and the relative intensity of bands. Multiple samples were pooled together in equal proportions based on their molecular weight and DNA concentrations. Pooled samples were purified using calibrated Ampure XP beads. Pooled and purified PCR product was used to prepare DNA library by following Illumina TruSeq DNA library preparation protocol. Paired-end sequencing was performed at Mr DNA (www.mrdnalab.com, Shallowater, TX, USA) on Illumina MiSeq device (Illumina Inc, San Diego, CA, USA) following the manufacturer’s guidelines. The sequences from each end were joined after quality q25 trimming of the ends with Mr DNA pipeline.

The resultant files were processed with Mothur v.1.36.1 [27]. The sequences were trimmed to remove primer and barcode sequences. Then, sequences that failed to comply with any one of the following criteria were excluded: average quality lower than 30 (window size of 50), minimum length of 200 bases, the presence of any ambiguities and more than 1 nucleotide mismatch to the primer. The sequences were then aligned using Mothur with the Silva reference database [28], and chimeras were detected with Uchime [29] and removed. Sequences were then taxonomically classified using the RDP reference database [30] with 80% confidence threshold. Sequences not classified into the Bacteria root, or classified in Chloroplast or Mitochondria were discarded.

The resulting alignment contained high-quality sequences ranging from 252 to 254 bp. Each sample was randomly subsampled to normalize to the same number of sequence (36,355). The normalized file was used as input for constructing the distance matrix and for clustering the sequences into operational taxonomic units (OTUs) at 3% dissimilarity cut-off. The sequences from the 10 samples are available at the NCBI Sequence Read Archive under the accession number SRP071254.

In order to increase the confidence in the generated clusters, all singletons and doubletons were removed, and the remaining clusters were used to calculate the species-richness estimators and the Shannon diversity index. The taxonomic assignment of the sequences and OTUs were performed as previously described and the graphics were constructed with the relative abundance of each taxon in the samples.

## Results

**Fig 1:**
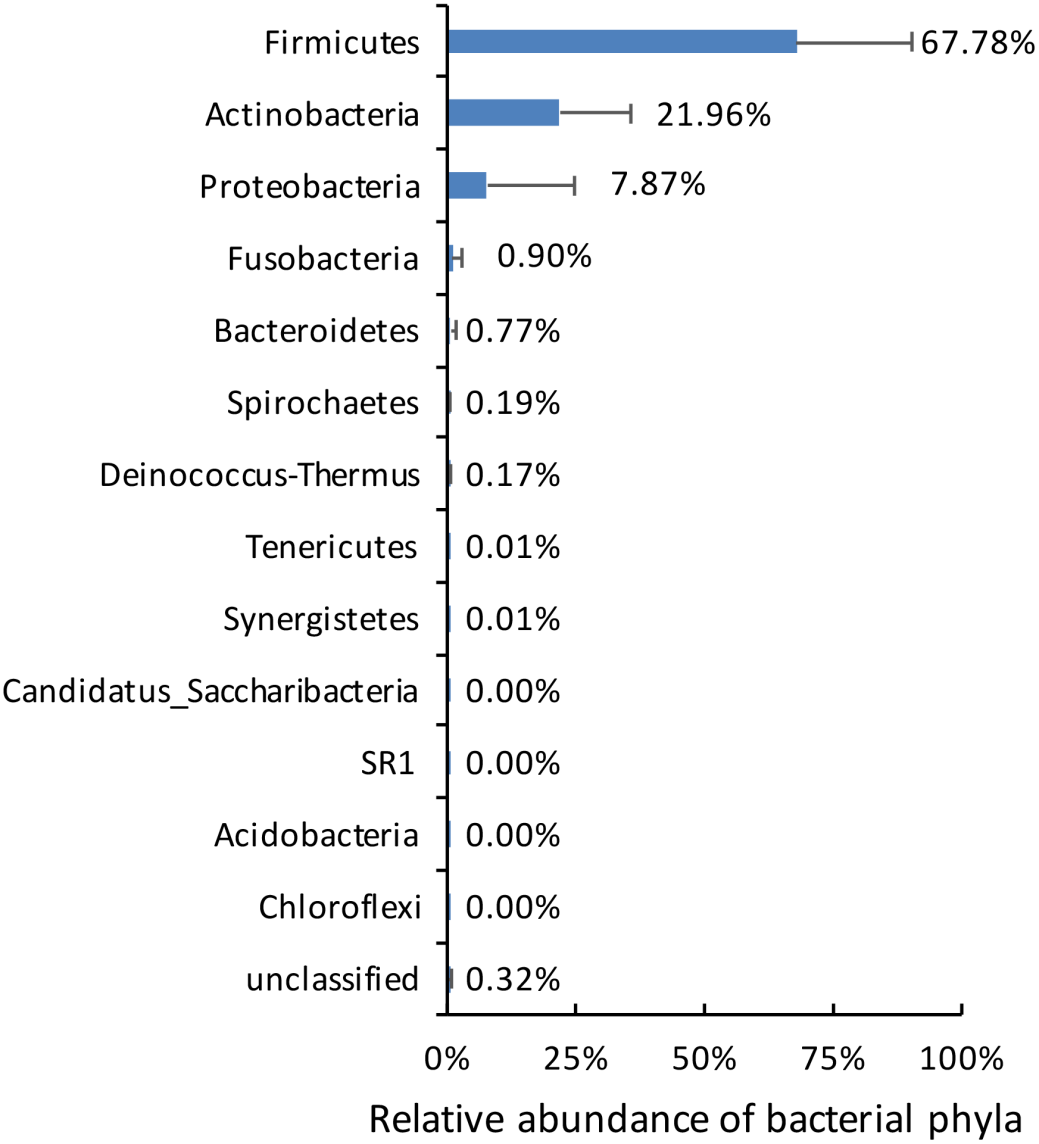
Average relative abundance of bacterial phyla composition in deep caries samples.

**Fig 2:**
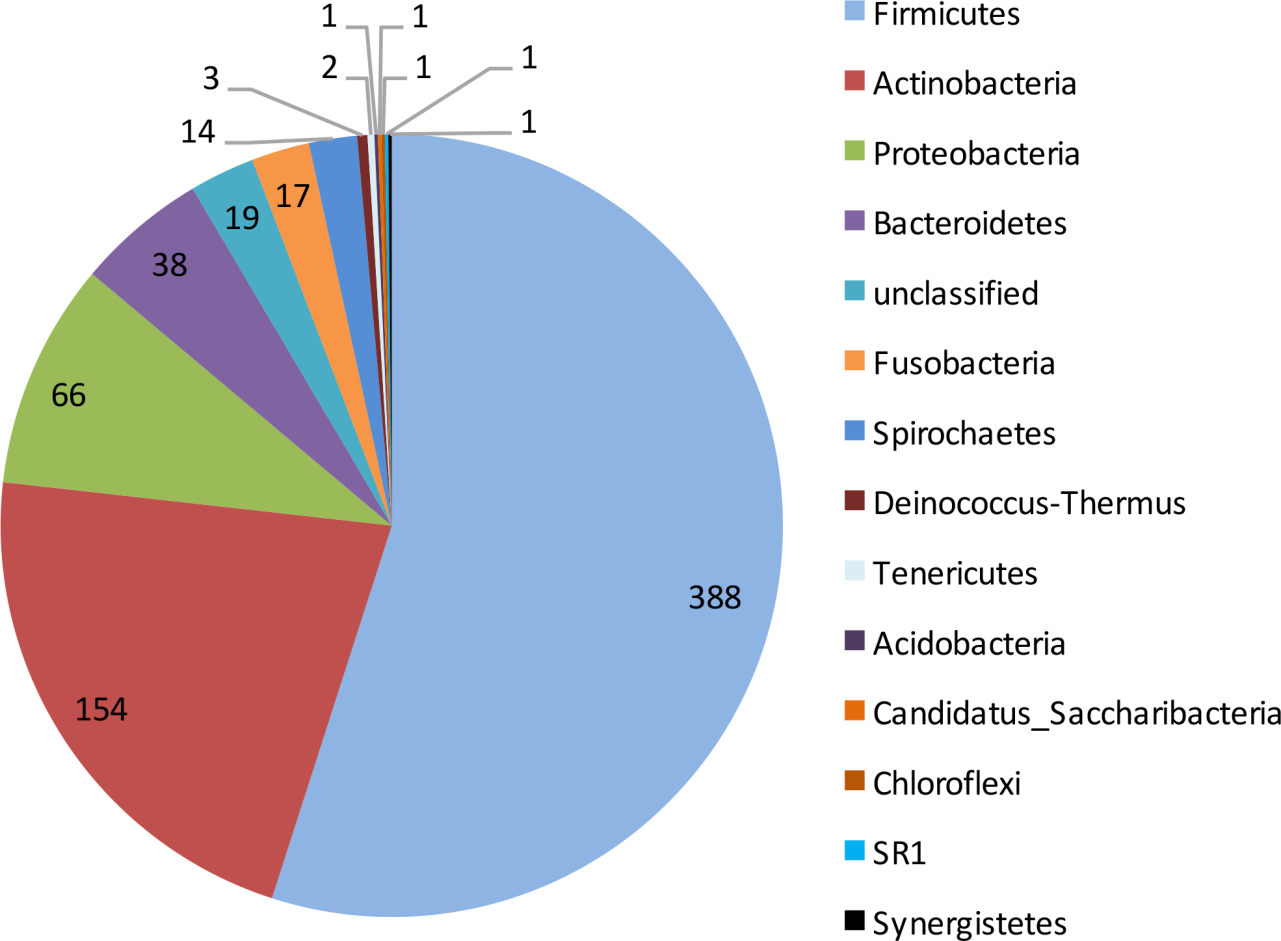
Taxonomic assignment (phylum level) of the 706 OTUs found in deep caries samples.

**Fig 3:**
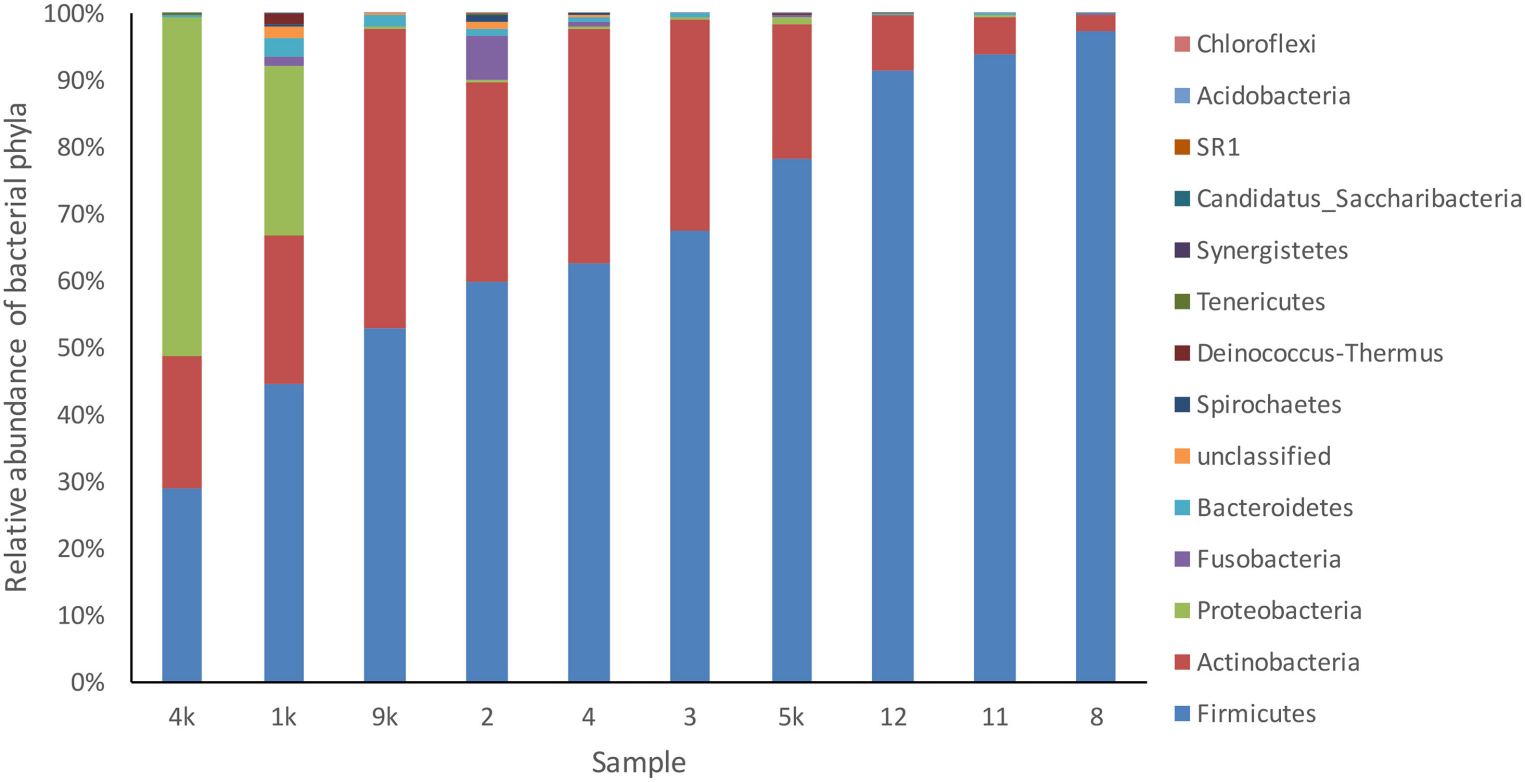
Relative abundance of bacterial phyla composition in deep caries samples.

Paired-end sequencing on Illumina MiSeq of dentinal caries samples revealed that all 10 cases were positive for the presence of bacterial DNA and resulted in 347,646 partial 16S rRNA gene sequences that passed the quality control. Bacterial taxa were mapped to 13 phyla and 101 genera composed by 706 different OTUs. Approximately 98% of the sequences belonged to 3 phyla: Firmicutes, Actinobacteria and Proteobacteria (Fig 1). Overall, the most represented phyla were Firmicutes (388 unique OTUs), Actinobacteria (154), Proteobacteria (66), and Bacteroidetes (38) (Fig 2). Nineteen OTUs could not be assigned to any bacterial phylum. Firmicutes was the most abundant phylum in 9 of the 10 samples; the other sample was dominated by Proteobacteria (Fig 3). Actinobacteria was the second most abundant phylum in 8 samples.

**Fig 4:**
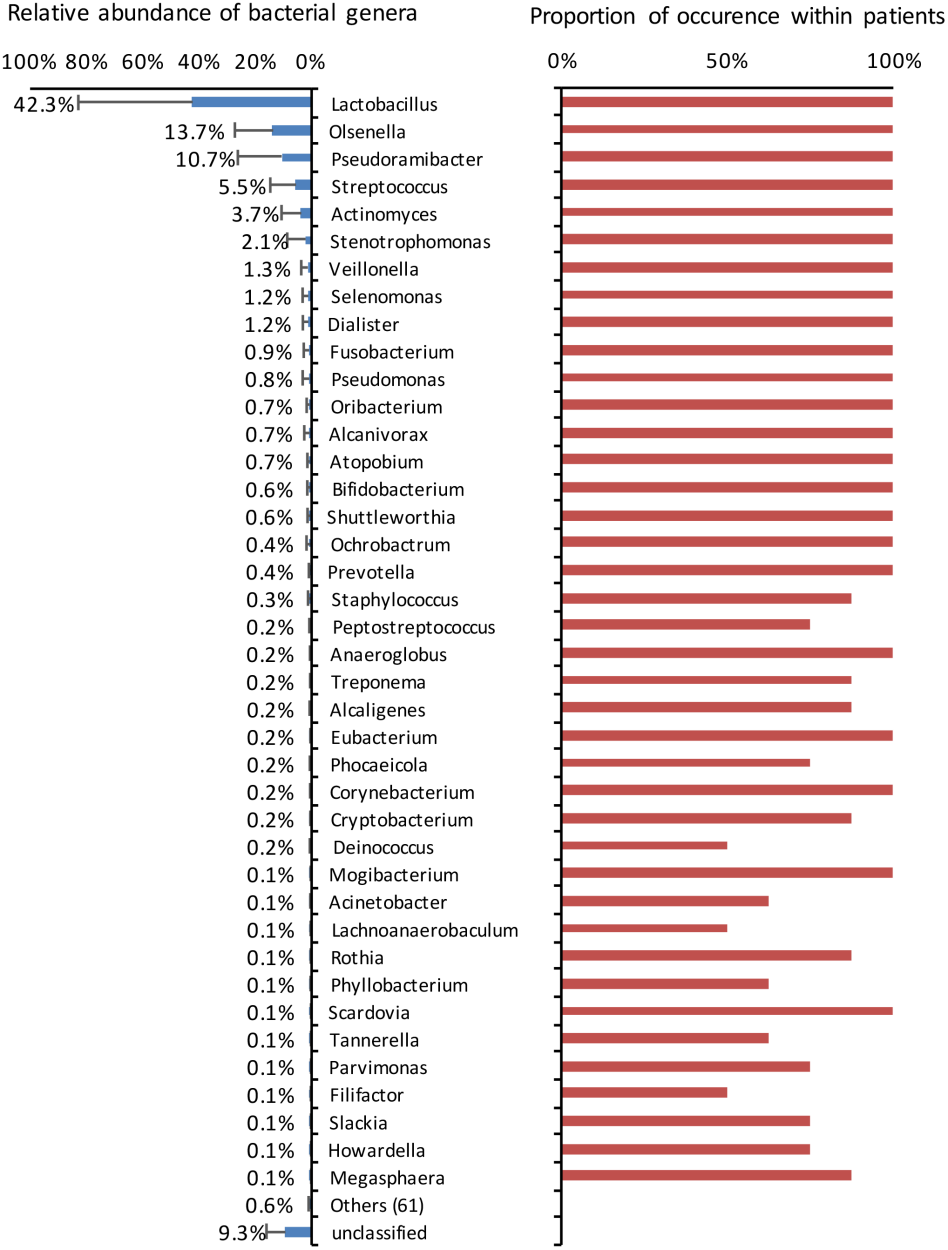
Average relative abundance of the top 40 most abundant bacterial genera (left bars), with the proportion of occurrence among the 10 deep caries samples (right bars).

**Fig 5:**
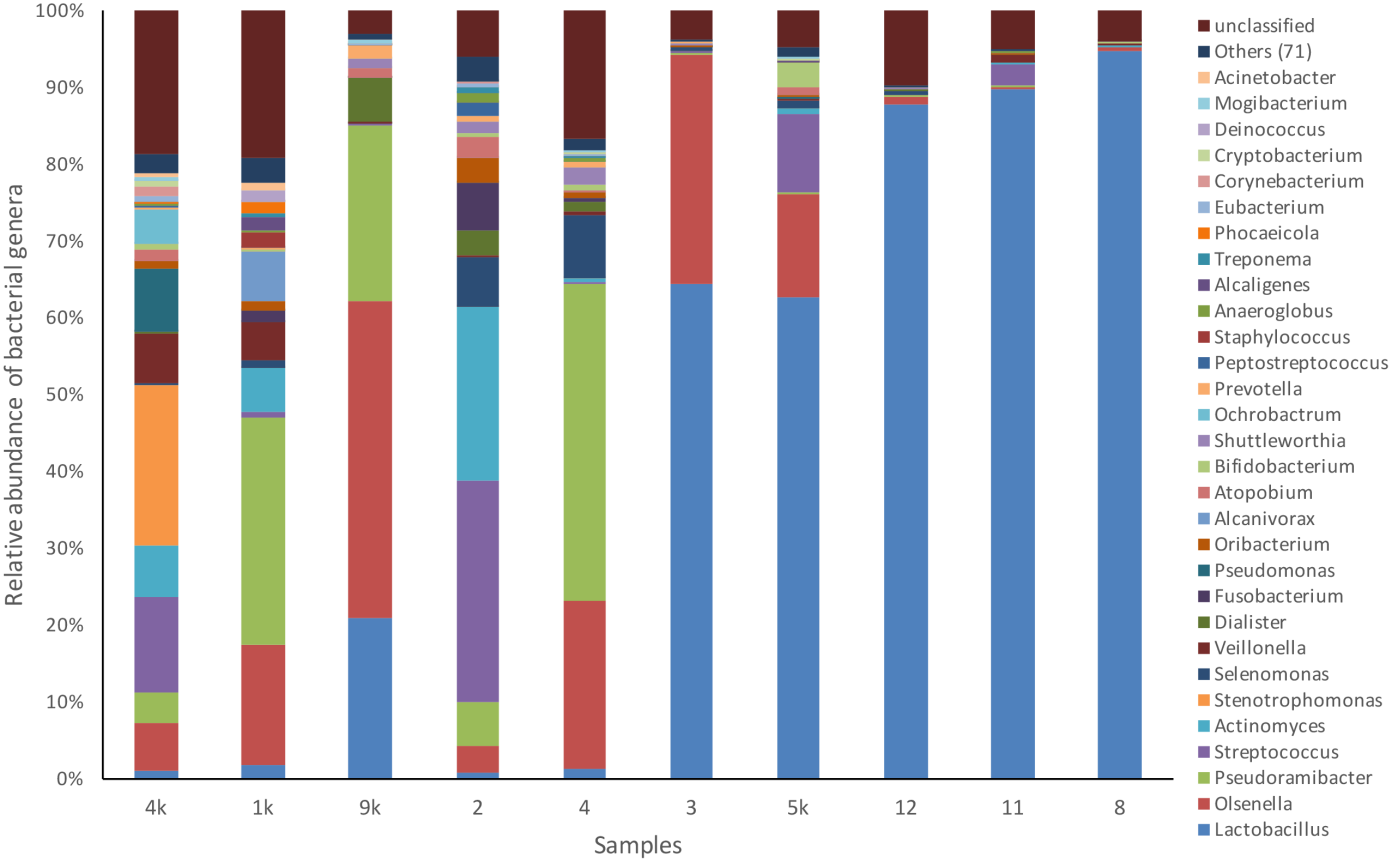
Relative abundance of the top 30 most abundant bacterial genera in each one of the 10 deep caries samples.

Several genera had representatives identified in all cases (Fig 4). Overall, the *Lactobacillus* genus accounted for 42.3% of the sequences, followed by *Olsenella* (13.7%), *Pseudoramibacter* (10.7%) and *Streptococcus* (5.5%). In terms of relative abundance per case, *Lactobacillus* was the most dominant genus in 5 teeth (ranging from 63% to 96% of the sequences), *Pseudoramibacter* in 2 teeth (29% and 43% of the sequences), and *Olsenella* (43%), *Streptococcus* (29%), and *Stenotrophomonas* (21%) in 1 tooth each (Fig 5). About 9% of the sequences could not be classified at the genus level.

Of the 6 most abundant OTUs, 4 belonged to the genus *Lactobacillus*, and the other two belonged to the *Olsenella* and *Pseudoramibacter* genera. The mean number of OTUs present per sample was 237 (range, 172 to 281); however, when taking into account only the OTUs composed by more than 10% of the sequences obtained in each case, the mean number of OTUs per case dropped substantially to 2.6 (range, 2 to 4).

**Fig 6:**
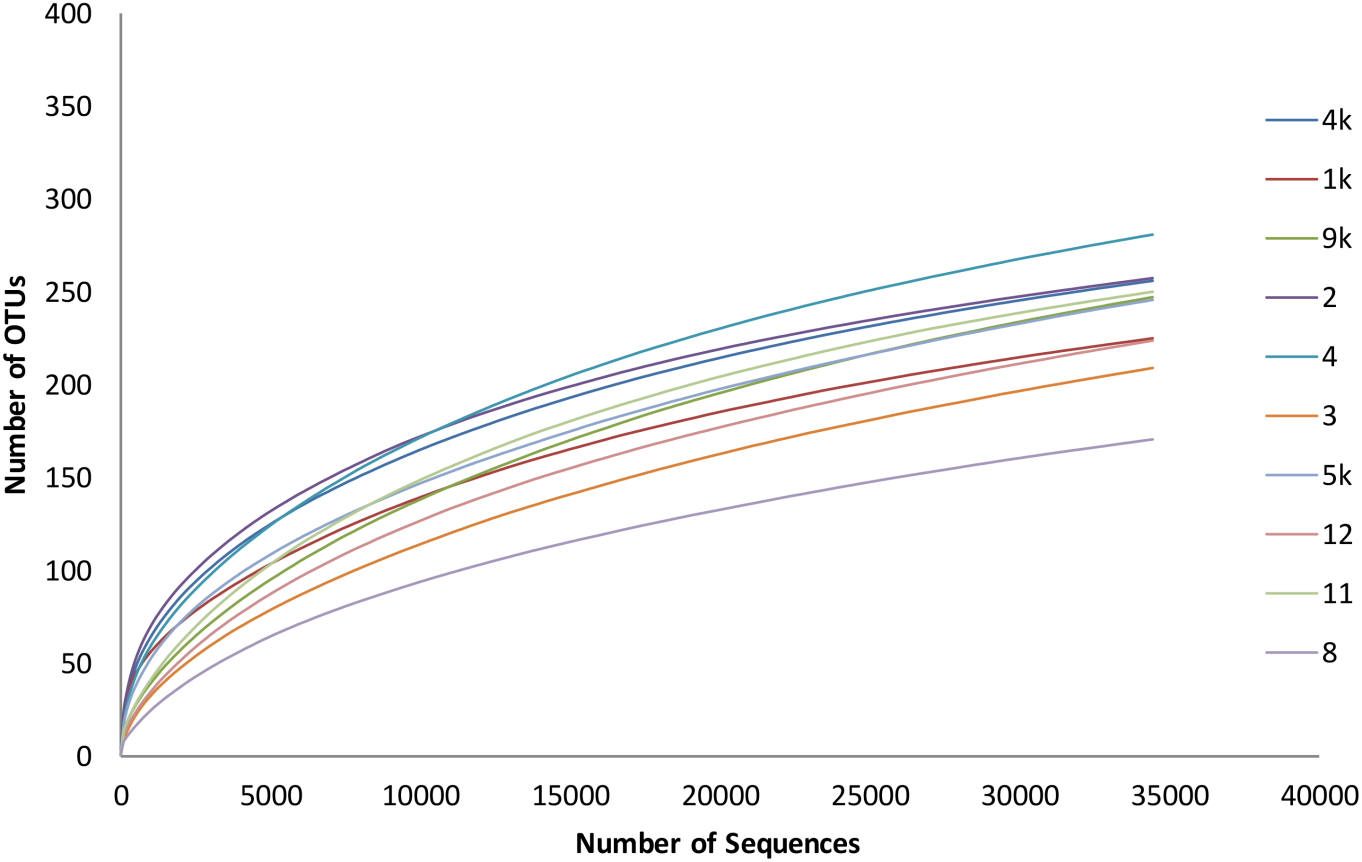
Rafefaction curve of the 10 deep caries samples.

Rarefaction curves are shown in Fig 6 and revealed a good coverage of the samples. Table 1 depicts data from diversity calculations. Chao and ACE nonparametric measures of richness revealed that there are a predicted mean of 333 and 361 species per dentinal caries sample, respectively. The Shannon index, wS^”#hich takes into account the species richness and evenness, was calculated per sample and is also shown in Table 1.

**Table 1:** Richness and diversity indexes of bacterial community from deep caries samples

Sample	OTUs	Chao	ACE	Shannon Index
4k	256	329	334	2.95
1k	226	299	300	2.73
9k	248	345	362	1.95
2	258	329	322	2.77
4	281	385	386	2.27
3	210	337	417	1.71
5k	247	354	442	2.20
11	251	314	334	2.06
12	225	352	340	1.85
8	172	285	377	1.35

## Discussion

Pulpitis comprises inflammation of the dental pulp and the major cause is the bacterial biofilm associated with caries. When the pulp tissue is directly exposed to the caries biofilm, an intense inflammatory response develops, leading to pulp tissue destruction and advance of infection. At this point, pulpitis is regarded as irreversible and pulp removal for root canal treatment is considered the most predictable approach to treat this condition and save the tooth. The bacterial species present in the advanced forefront of the carious biofilm are arguably the ones involved with the etiology of pulpitis and the first ones to invade the pulp tissue to cause endodontic infection. The priority of this study was to deal with the microbiome of dentinal caries lesions related with symptomatic irreversible pulpitis using a NGS technology.

Half of the advanced caries lesions were highly dominated by lactobacilli, which comprised from 63% to 96% of the sequences identified in these samples. Previous studies using culture and molecular methods have also observed that dentinal caries lesions can be classified according to the relative abundance of *Lactobacillus* [10, 19, 31]. A pyrosequencing study showed that *Lactobacillus* was one of the 8 genera that increased significantly in cavitated dentin lesions [20]. Another study using pyrosequencing to evaluate the microbiota associated with dentinal caries revealed that *Lactobacillus* species were one of the most abundant, and the species *L*. *fermentum*, *L*. *rhamnosus* and *L*. *crispatus* were dominant in acidic areas of the carious dentin [21]. Lactobacilli are acidogenic and aciduric bacteria that have been strongly associated with caries. They are not high producers of extracellular polysaccharides [32] and, because of their low adherence to smooth tooth surfaces, lactobacilli are more frequent in retentive areas, such as cavities [33]. It has been proposed that lactobacilli are involved more in the progression of caries rather than the initiation [34]. Very few other bacterial taxa were detected in the samples dominated by lactobacilli, suggesting that in some way the latter may have been favored by the changed environmental conditions. Actually, establishment of an acidic environment diminishes the bacterial diversity in caries lesions and favors the establishment of aciduric bacteria such as lactobacilli [35].

In the other half of cases, which showed low levels of lactobacilli, the most dominant genera were *Pseudoramibacter* in 2 teeth, and *Olsenella*, *Streptococcus*, and *Stenotrophomonas* in one tooth each. Most of these taxa have been frequently detected in dentinal caries by previous studies [12, 14, 20–21, 25, 31]. Pyrosequencing studies have reported a high dominance of some of these genera, especially *Olsenella* and *Streptococcus*, in dentinal caries lesions [20–21]. The degree of dominance of these taxa was inferior when compared to the *Lactobacillus* levels in the other five samples, and in all of these cases several other taxa were also present in a typically mixed community. The reasons why lactobacilli are highly dominant in some cases and virtually absent in others remain elusive. If this difference in the relative abundance of lactobacilli in caries lesions of teeth with pulpitis is associated with any clinical condition such as pain requires evaluation by further studies, since all cases examined herein were symptomatic. Moreover, a larger sample size may be necessary to reveal associations with any clinical and demographic conditions.

The possibility also exists that the difference in lactobacilli abundance may represent a transition from a cariogenic microbiota to one that participates in the initial infection of the pulp tissue. Shifts in the composition of the microbiota may be caused by ecological changes in the affected tissues. Whereas bacteria from the superficial layers of caries have the host diet, usually rich in sugars, as an important source of nutrients, bacteria occurring at the deepest layers of caries may face a rather different condition. Pulp inflammation may represent another source of nutrients, especially in the form of proteins from the inflammatory exudate, which favor and select asaccharolytic and proteolytic bacteria. In addition, the immune host defenses can also exert a selective pressure on the biofilm composition. The hypotheses of a shift in the microbiota may find support in the fact that while lactobacilli are rarely found in infected canals, the other bacterial taxa found in deep caries samples with low lactobacilli levels are frequently identified in endodontic infections [36–40]. This requires confirmation by further studies.

Curiously, *Prevotella* species, which have been reported to be one of the dominant taxa in deep dentinal caries lesions by previous studies [14, 18–19, 21], were found in very low levels in the present one. Although *Prevotella* species occurred in all cases, they were always below 1% of the community, except for 1 case (2%). Methodological issues may contribute to this discrepancy, but this is also possibly related to geographical differences in the composition of the oral microbiota. For instance, a study of endodontic infections showed that *Prevotella* species were much more prevalent in samples from USA patients than Brazilian individuals [41].

At a higher taxonomic level, the most abundant and diverse phylum was Firmicutes, followed by Actinobacteria. This is in agreement with a previous study using pyrosequencing [21]. Firmicutes were mostly represented by *Lactobacillus*, *Pseudoramibacter* and *Streptococcus*, 3 of the 5 most dominant genera, while *Olsenella* and *Actinomyces*, the other dominant genera, were the most common representatives of the *Actinobacteria* phylum. These two phyla accounted for 90% of the sequences detected in this study, which shows their strong association with deep dentinal caries lesion and pulp inflammation.

NGS technologies permit massive DNA sequencing with a much higher throughput than the conventional Sanger sequencing approach, and have been widely used to unravel the human microbiome in diverse oral and nonoral sites [36, 39, 42–47]. The present study revealed a much larger bacterial diversity and complexity in advanced caries lesions than demonstrated by previous culture and molecular microbiology studies and is in agreement with recent studies of the dentinal caries microbiome using NGS technologies [19–21]. An important limitation of this and the large majority of previous studies using NGS technologies is that only short 16S rRNA gene sequences are used for identification. To increase the confidence in taxonomic assignment, we used the Naïve Bayesian supervised classification method, which is better than the clustering approach based on sequence similarity, and a high cutoff threshold (80%). Even so, data are restricted to identification to the genus level and above.

In conclusion, this study showed a high bacterial diversity in deep dentinal caries lesions associated with symptomatic irreversible pulpitis. The present findings are very similar to those of previous studies using culture and molecular methods to evaluated deep dentinal caries lesions from teeth without pulpitis. The microbiota could be classified according to the relative abundance of *Lactobacillus* species. Except for *Lactobacillus* species, most of the highly prevalent and abundant bacterial taxa identified in this study have been usually detected in infected root canals. This suggests that, in addition to being involved with pulp damage and inflammation, deep dentinal caries lesions may be the primary source of bacteria for endodontic infections. The information generated is an important contribution to the knowledge of bacterial taxa that cause irreversible pulpitis and participate in the early stages of pulp infection. Recognition of the bacterial agents involved with pulpitis may also lead to therapeutic insights and strategies to deal with the initial pulp infection in an attempt to make the conservative pulp treatment more predictable, reducing the need of removing the pulp for root canal treatment.
